# The Role of Consciousness in Coupling Emotions, Motivations, and Behaviors

**DOI:** 10.3390/brainsci14060582

**Published:** 2024-06-05

**Authors:** Sergio Frumento, Danilo Menicucci

**Affiliations:** Department of Surgical, Medical, Molecular and Critical Area Pathology, University of Pisa, 56126 Pisa, Italy; sergio.frumento@med.unipi.it

## 1. An Introduction and the Rationale of this Special Issue: The Neglected Purpose of Consciousness

A potential function of consciousness is to integrate emotions, motivations, and subsequent behaviors into a coherent narrative [1]. Even if it is intuitive, this hypothesis is partially challenged by evidence that aversive stimuli can elicit consistent behaviors even when not consciously perceived [2] or that individuals with arachnophobia instinctively avoid spiders even knowing that they are virtual [3]. Given this information, how might consciousness be involved in linking emotions, motivations, and behaviors? Can this mechanism fail under some conditions?

To investigate the possible role of consciousness in this coupling, a tentative approach involves examining the effect of consciousness alterations in each relevant construct. This goal can be pursued through a variety of frameworks; for example, by decreasing phobic symptoms by rewarding the spontaneous activation of phobia-related brain areas [4,5], addressing the irrational motivations that induce people to indulge in risky behaviors despite massive awareness campaigns promoted in this regard [6], or trying to replicate the conscious–unconscious relationship in artificial intelligence (AI) systems [7].

## 2. Overview of Published Articles

This Special Issue captures a variety of frameworks in which the role of consciousness can be ascribed when considering the coupling of emotions, motivations, and behaviors. The majority of papers reported empirical data ranging from behavioral to electrophysiological correlates integrated with self-report measures; others introduced and discussed computational models.

The article by Lee et al. investigated how an altered state of consciousness induced by a meditation-based protocol (Awareness Training Program, ATP) affected self-reported psychological stress and age-related EEG correlates; they found that the ATP reduced stress and made the EEG correlates more similar to those of a younger population.

In a task involving the recognition of various emotions and of AI/real content, Tarchi et al. found EEG correlates already differentiating emotional from non-emotional and authentic from AI-generated faces in the early stages (e.g., P100, 90–140 ms) of stimulus processing.

Analogously, Hou et al. found early (e.g., N1, 80–120 ms; P1, 140–180 ms) EEG correlates differentiating more- from less-attractive faces administered at both supraliminal and subliminal levels.

These unconscious effects on behavior were also studied by Panteli et al., who evaluated the relationship between emotion regulation/awareness and externalizing problems (e.g., problematic behaviors derived from poor impulse control) in preadolescents. Both physiological and subjective data supported the idea that greater proneness to externalizing problems is correlated with weak regulation and awareness of one’s own emotions (as well as their physiological correlates).

Similarly, Cipriani et al. suggested that interoceptive sensitivity could modulate awareness of changes in climate, providing a detailed discussion of the psychophysiological mechanisms underlying these factors. The capability to detect external as well as internal signs of climate change could underlie differences in feeling (and actively trying to reduce) the risks it implies.

The *entwined hypothesis* described by Grindrod and Brennan proposes that interoceptive and emotional awareness can regulate the rapidity and accuracy of cognitive processes; this revisitation of LeDoux’s two-pathways theory of emotions and of Kahneman’s “thinking fast, thinking slow” approach to cognition also carries implications for artificial intelligence.

Reviewing the scientific literature on the Projective Consciousness Model (PCM), Rudrauf et al. stress the relevance that the point of view taken by an agent perceiving internal or external stimuli can have on their motivation and hence behaviors.

## 3. Conclusions and Future Directions

The most recurrent topics mentioned in the papers collected in this Special Issue concern the irrelevance (or, at least, the unnecessity) of conscious awareness when performing complex tasks (such as discriminating AI-generated from authentic stimuli) and potentially impactful choices (e.g., evaluating a face’s attractivity) in favor of the relevance of interoceptive sensitivity (e.g., in shifting toward more or less pro-environmental behaviors).

This evidence is in agreement with the widespread adoption of mindfulness-based practices as good practices for improving psychological well-being by leveraging improvement in proprioceptive awareness.

In addition, from studies on relationships between interoception and consciousness in this Special Issue, we foresee interesting future perspectives on the development of artificial intelligence (AI). Indeed, there is a growing scientific interest in (1) giving a corporeity to AI, for example, by providing it with a physical and (even if mechanical) sensible body [8] able to replicate the peripherical–central information flux occurring in the physiology supporting human consciousness [9,10] and in (2) adding a subconscious or unconscious layer to AI structures currently corresponding to a potentially infinite global workspace [7]. Both these research paths could benefit from the results described in the relevant articles published in this Special Issue.

In conclusion, in our opinion, a final message can be derived from the contributions to this Special Issue: *knowing* something and *deeply feeling it and its consequences* are processes that can be surprisingly different and mutually unrelated; this notion will gain increasing relevance as humanity’s future challenges—climate change and artificial intelligence—move toward unprecedented scenarios.
